# RPNs Levels Are Prognostic and Diagnostic Markers for Hepatocellular Carcinoma

**DOI:** 10.1155/2022/7270541

**Published:** 2022-08-29

**Authors:** Wangyang Zheng, Yuling Zheng, Xue Bai, Yongxu Zhou, Liang Yu, Daolin Ji, Kaiming Leng, Nanfeng Meng, Hang Wang, ZiYue Huang, Yi Xu, Yunfu Cui

**Affiliations:** ^1^Department of Hepatopancreatobiliary Surgery, Second Affiliated Hospital of Harbin Medical University, Harbin 150086, China; ^2^The Key Laboratory of Myocardial Ischemia, Harbin Medical University, Ministry of Education, Harbin 150086, China; ^3^Department II of Gastroenterology, Third Affiliated Hospital of Harbin Medical University, Harbin 150086, China; ^4^Second Affiliated Hospital of Harbin Medical University, Department of Pediatric, Harbin 150086, China; ^5^Department of Clinic of Internal Medicine, Ulm University, Ulm, Germany; ^6^Department of Hepatopancreatobiliary Surgery, Fourth Affiliated Hospital of Harbin Medical University, Harbin 150086, China; ^7^Department of Hepatopancreatobiliary Surgery, Qingdao Municipal Hospital, Qingdao, China; ^8^Department of Pathology, Li Ka Shing Faculty of Medicine, The University of Hong Kong, Hong Kong China

## Abstract

The ribophorin family (RPN) is an essential regulatory subunit of the proteasome. By influencing the ubiquitin-proteasome system activity, ribophorins (RPNs) are responsible for almost all physiology and pathology processes of mammalian cells. Nevertheless, little is known about the role of RPNs in HCC. In this work, we first evaluated the transcriptional levels and the prognostic and diagnostic value of RPNs based on the public database. Firstly, we found all RPNs were surprisingly consistently upregulated in HCC tissues. Moreover, the RPNs' expression pattern is correlated with HCC tumor grade. The TCGA HCC platforms' data indicated that RPN2, RPN3, RPN6, RPN9, RPN10, RPN11, and RPN12 have robust diagnosis values. Then, survival analysis revealed that the high expression of RPN1, RPN2, RPN4, RPN5, RPN6, RPN9, and RPN11 was correlated with unfavourable HCC overall survival. Then, genetic alteration, immune infiltration feature, gene-genes network, and functional enrichment for RPNs indicated that RPNs have many potential biosynthesis activities expert for UPS functions. Moreover, western blot and qRT-PCR results confirmed these results. The silencing of RPN6 and RPN9 significantly reduced HCC cells' proliferation, migration, and invasion ability in vitro. An in vivo tumor model further validated the oncogene effect of RPN6 on HCC cell growth. Moreover, RPN6 and RPN9 could promote cell migratory and invasive potential by affecting the epithelial-mesenchymal transition (EMT) process. In summary, this study suggests that the RPN family has the potential to be potential biomarkers and targets for HCC.

## 1. Introduction

Hepatocellular carcinoma (HCC) ranks as the fifth most common cancer and the second cause of cancer-related death worldwide [1, 2]. In the last two decades, the incidence of HCC has been increasing globally while even doubling in the United States [3]. Although surgery has become a standard treatment for HCC, most patients have reached the late stage when being diagnosed and are not surgical candidates [4, 5]. The five-year survival rate for patients with HCC is only 7%. Therefore, it is urged to find new biomarkers for the detection, diagnosis, and prognosis and new targets for molecule therapy [6].

The ubiquitin-proteasome system (UPS) is highly conserved in eukaryotic cells [7]. UPS is responsible for almost all cellular progress, functioning as degrading cellular proteins. Incorrect protein degradation may give rise to many diseases, including cancers, in versatile ways, reported in proliferation, autophagy, and drug resistance [8–10]. The Ribophorin family have 14 members: RPN1, RPN2, RPN3/PSMD3, RPN4/PSMD9, RPN5/PSMD12, RPN6/PSMD11, RPN7/PSMD6, RPN8/PSMD7, RPN9/PSMD13, RPN10/PSMD4, RPN11/PSMD14, RPN12/PSMD8, RPN13/ADRM1, and RPN14/PAAF1. They construct the 19 s (regulatory particles) of the 26 s proteasome. Some subunits (RPN1, RPN10, and RPN13), their specificity structure, and location even determine whether proteasomes work or not and which protein should be degraded [11]. Therefore, many RPNs have been deregulated and have robust oncogene functions in cancers [12–15]. Moreover, some studies reported that RPNs' expression increased after virus infection [16]. As we all know, most HCC patients are initiated by chronic liver hepatitis virus B or C infections. Therefore, RPNs may do something special in HCC pathology [17]. Many cancer targets and new biomarkers have been identified and verified [18, 19]. This study extended the knowledge on RPNs and HCC to appraise distinct prognostic values, predict potential functions and putative targets, and then evaluate by experiments.

## 2. Materials and Methods

### 2.1. Specimens and Cell Lines

Between January 2016 and January 2018, 54 HCC specimens and corresponding non-cancerous tissues were harvested from patients at the Second Affiliated Hospital of Harbin Medical University. These fresh specimens were preserved in liquid nitrogen. None of the patients received radiotherapy or chemotherapy before surgery. This study was authorized by the Ethics Committee of the Second Affiliated Hospital of Harbin Medical University. Hcclm3, Huh7, and Wrl-68 cells were obtained from the Cell Bank of the Chinese Academy of Sciences (Shanghai, China). Hcclm3 and Huh7 were maintained in DMEM (Gibco, Grand Island, NY, USA) containing 10% fetal bovine serum (Invitrogen Life Technologies, Carlsbad, CA, USA), while Wrl-68 cells in RPMI-1640 (Gibco, Grand Island, NY, USA). They were all cultured in an atmosphere of 37°C with 5% CO2.

### 2.2. Cell Transfection and RNA Isolation

Small-hairpin RNA directed against RPN6 and RPN9 were designed and synthesized (Gene Chem, Shanghai, China). Their sequences are listed in Table S1. An empty Sh-NC vector was used as a control. The procedure of lentiviral infection was conducted by the instructions of the manufacturer. The selection of qualified cells was performed using puromycin for 3-4 weeks. TRIzol (Sigma, MO, USA) was used for total RNA isolation in HCC tissue specimens and cultured cells. Then, we checked the many features such as nucleic acid concentration, OD230, OD260, and OD280 of RNAs.

### 2.3. QRT-PCR and Western Blotting

RNAs were applied to synthesize the complementary DNA (cDNA) with a First Strand cDNA Synthesis Kit (Roche, Germany). RT-qPCR was carried out using the FastStart Universal SYBR Green Master Kit (Roche, Germany). The primer sequences are listed in Table S1. GAPDH was used for the internal control of RNA expression. Sequences of all of the gene primers are listed in Table S1. The mRNA fold change data were normalized and calculated using equation (2) ^−△△^CT. Western blot was carried out following standard protocols as previously described [20].

### 2.4. Tumor Xenograft Study

Hcclm3 cells were transfected with sh-RPN6 or the sh-NC control. After collecting these cells, 3 × 10^6^ cells were injected subcutaneously into either side of female BALB/*c* nude mice (6–8 weeks of age, *n* = 3 per group). Tumor growth was measured, and tumor volumes were calculated every three days. The mice were euthanized 18 days after injection, and the tumors weights were measured.

### 2.5. CCK-8 and Wound Scratch Assay

CCK-8 (Cell counting kit-8) (Dojindo, Japan) was employed to determine cell viability. A density of 4 × 10^4^ cells per well was seeded in 96-well plates. Ten *μ*l of reagent was added to each well and maintained for two h at 37°C. At 0, 24, 48, 72, and 96 h, the cells were measured by the reader (Tecan, Switzerland) at a wavelength of 450 nm. Cell motility was measured by wound scratch experiments. Using a 200 *μ*L pipette tube, we created an acellular area. Then, cells were placed in a serum-free DMEM medium. The area was measured at 0, 24, and 36 h.

### 2.6. Migration and Invasion Assays

Coated with Matrigel (for invasion) or not (for migration), transwell chambers (Corning, New York, USA) were applied to further access cellular motility. 5 × 10^4^ cells were resuspended in 200 *μ*L of FBS-free DMEM in the higher chambers while lower chambers were placed with 600 *μ*L 10% FBS DMEM. After incubating for 48 h at 37°C, cells on the upper surface of the chambers were eliminated. Then, cells passed through the membranes were fixed and stained. The numbers of invasive or migrated cells were counted using a microscope.

### 2.7. ONCOMINE Analysis

To analyze the relative expression of RPNs in a variety of tumors, ONCOMINE, a free open-accessin-depth bioinformatic database (https://ONCOMINE.org), was used, including 715 datasets and 86,733 samples. The mRNA expression profiles of RPNs in HCC were confirmed by this database, using a students' *t*-test to generate a *p*-value.

### 2.8. UALCAN Database

UALCAN (https://ualcan.path.uab.edu) is an easy-to-use online tool to analyze online microarray data from the TCGA databases. Besides, they also provide some clinical features and survival prognosis data based on gene expression differences in 31 cancer types. In our study, we analyzed the expression pattern of RPNs in HCC and the relationships between RPNs' mRNA expression pattern and patients' tumor grades. Moreover, we used Bonferroni correction to test the *p-*value significance.

### 2.9. UCSC Xena

UCSC Xena (https://xena.ucsc.edu/) is a free and open-access online tool for getting homogenized data from multiple databases, including TCGA, ICGC, TARGET, GTEx, and CCLE [21]. The database provides information on copy number, methylation, somatic mutation, gene expression, protein expression, and clinical data.

### 2.10. Kaplan–Meier Plotter Database

The Kaplan–Meier Plotter (https://kmplot.com/analysis/) is an online tool established by TCGA patient survival information and is used to evaluate the prognostic value of RPNs in HCC [22]. The prognostic value was evaluated by four indexes, OS (overall survival), PFS (progression-free survival), RFS (recurrence-free survival), and DSS (disease-specific survival).

### 2.11. Cbioportal Analysis

The Cbioportal database (https://www.cbioportal.org/) is an open-access platform which provides interactive exploration of multidimensional Cancer Genomics data [23]. We analyzed the genetic alterations of the RPN family genes in HCC based on the 8 studies containing 1308 samples. Genomic profiles, mutations, survival data, and mRNA expressions were mentioned for analyzing the 14 RPN family genes.

### 2.12. GeneMANIA and Functional Enrichment Analysis

GeneMANIA (https://www.genemania.org) is an online platform for deriving hypothetical genes interaction networks based on those locations or functions. By querying a list of genes, GeneMANIA can generate multiply genes with similar functions and illustrate the relationship between the queried gene set and the dataset by constructing an interactive network [24]. This study used GeneMANIA to build a gene-gene interaction network for RPNs. We used the STRING database to perform the functional enrichment analysis [25].

### 2.13. Statistical Analysis

We used GraphPad Prism 5.01 software and SPSS 22.0 to analyze the data in this study. The diagnosis value of RPNs was measured by ROC curves, which were plotted using the SPSS software. Quantitative data were expressed as mean ± S.D.

## 3. Results

### 3.1. All RPNs' Transcriptional Levels Are Extremely High in Cancer Tissues

**Figure 1:**
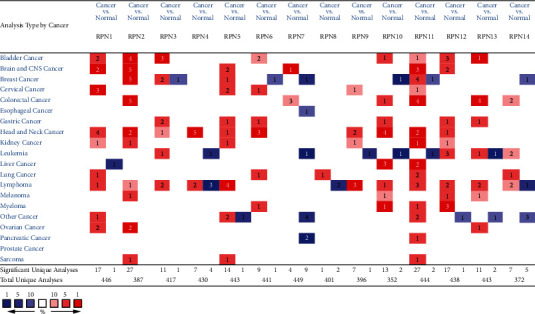
The transcription levels of RPNs in pan-cancers (ONCOMINE).

We first analyzed all of the RPN1-RPN14 mRNA expression levels in multiple human cancers using the Oncomine database (Figure 1). The analysis showed that the transcriptional levels of all RPNs were upregulated in human cancers. Some RPNs, such as RPN10 and RPN11, were upregulated in HCC patients.

### 3.2. Relationship between the Transcriptional Levels of RPNs and the Clinicopathological Parameters of HCC Patients

**Figure 2:**
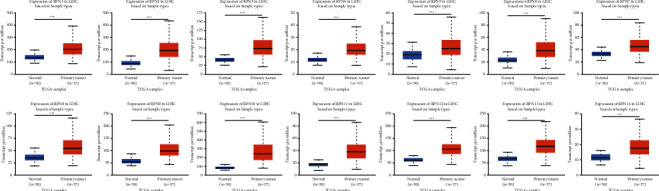
RPNs are differentially expressed between HCC and normal tissues (UALCAN). ^*∗*^*p* < 0.05, ^*∗∗*^*p* < 0.01, and ^*∗∗∗*^*p* < 0.001.

**Figure 3:**
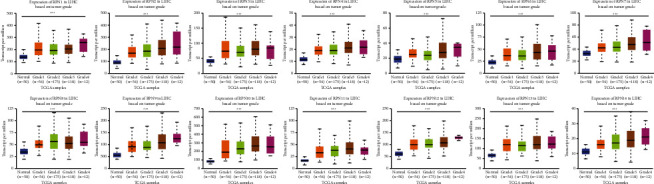
Correlation between the levels of 14 RPNs and HCC patient clinicopathological characteristics (UALCAN). ^*∗*^*p* < 0.05, ^*∗∗*^*p* < 0.01, and ^*∗∗∗*^*p* < 0.001.

Then, we compared the RPNs' transcriptional levels in HCC with those in normal samples using the UALCAN database (Figure 2). All RPNs were surprisingly upregulated in HCC tissues than normal, with a low *p*-value. After Bonferroni correction, the *p*-value of all RPNs is still lower than the corrected (*p* < 0.003571). Moreover, we examined our findings in the ONCOMINE database (Table 1). In Roessler's dataset, RPN1 was upregulated with a fold change of 1.249 (*p*=1.09*E* − 14), RPN2 with a fold change of 1.713 (*p*=1.54*E* − 74), RPN3 with a fold change of 1.286 (*p*=7.37*E* − 17), RPN4 with a fold change of 1.239 (*p*=7.50*E* − 11), RPN5 with a fold change of 1.692 (*p*=5.83*E* − 37), RPN6 with a fold change of 1.654 (*p*=3.73*E* − 36), RPN7 with a fold change of 1.465 (*p*=1.02*E* − 23), RPN8 with a fold change of 1.162 (*p*=9.26*E* − 6), RPN9 with a fold change of 1.458 (*p*=4.73*E* − 29), RPN10 with a fold change of 2.265 (*p*=2.10*E* − 85), RPN11 with a fold change of 2.243 (*p*=4.71*E* − 74), RPN12 with a fold change of 1.543 (*p*=7.28*E* − 31), RPN13 with a fold change of 1.288 (*p*=1.40*E* − 19), and RPN14 with a fold change of 1.131 (*p*=3.89*E* − 5), which is in accordance with our finding [26]. Next, using the UALCAN database, we analyzed whether the RPNs' transcriptional levels were related to clinicopathological characteristics (Figure 3). As shown in Figure 3, when tumor grade increases, the RPNs' expression pattern also increases. The mRNA levels of all RPNs were positive for tumor differentiation with *p* < 0.05. That means the measurement of RPNs' levels may help determine the patient's tumor stage.

### 3.3. Diagnosis and Prognostic Significance of the RPNs in HCC

**Figure 4:**
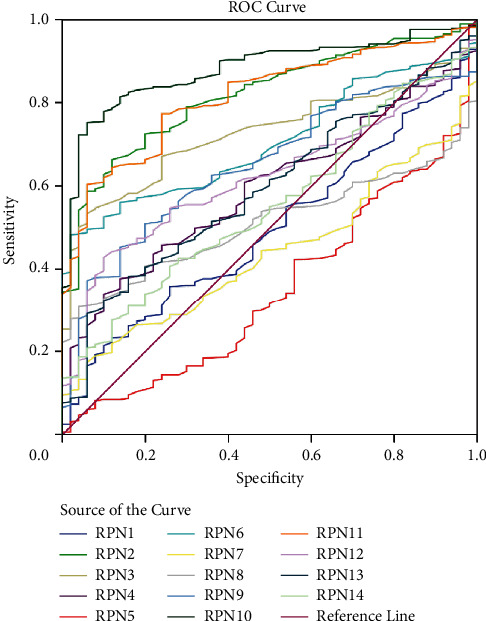
The ROC curve for analyzing of the 14 RPNs' diagnostic value.

To evaluate the value of RPNs in the diagnosis of HCC, the computing receiver operating characteristic (ROC) analysis was used. Using the data obtained from the HCC platform from the UCSC website, we draw the ROC curves to analyze the RPNs' diagnostic significance. As the figure showed (Figure 4), RPN2 (Area = 0.818 and *p* < 0.00011), RPN3 (Area = 0.728 and *p* < 0.0001), RPN6 (Area = 0.704 and *p* < 0.0001), RPN9 (Area = 0.650 and *p* < 0.0001), RPN10 (Area = 0.880 and *p* < 0.0001), and RPN11 (Area = 0.815 and *p* < 0.0001) were all helpful for HCC diagnosis. Our finding suggested that RPN2, RPN3, RPN6, RPN9, RPN10, RPN11, and RPN12 have the potential to be useful as biomarkers for HCC.

**Figure 5:**
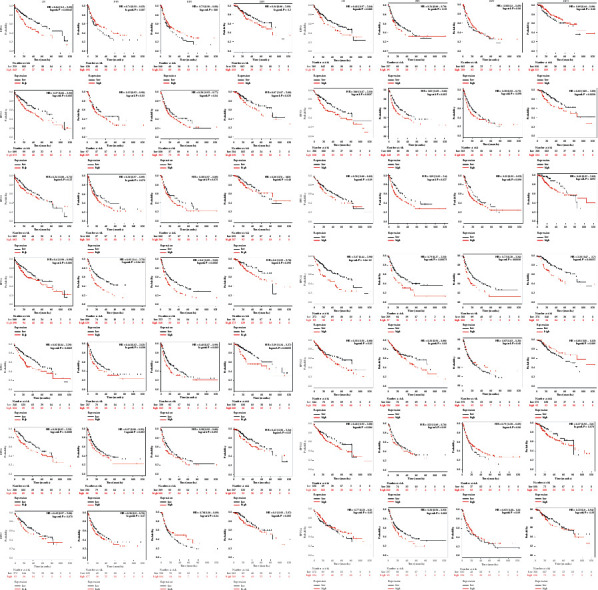
Prognostic value of the mRNA expression for distinct RPNs in HCC (Kaplan–Meier Plotter).

Then, we evaluated the prognostic role of RPNs in HCC using the Kaplan–Meier Plotter databases (Figure 5). In the OS (overall survival) group, lower RPN1, RPN2, RPN5, RPN6, RPN9, and RPN11 expressions indicated better survival. In the PFS (progression-free survival) group, reduced RPN4, RPN5, and RPN11 were correlated with better survival. In the RPS (recurrence-free survival) group, RPN4, RPN5, RPN9, RPN10, and RPN11 downregulation were connected with better survival. In the DSS (disease-specific survival) group, the downregulation of RPN2, RPN5, RPN9, and RPN11 may indicate better survival. These findings were consistent with the UALCAN analysis and suggested that some RPNs such as RPN5, RPN9, and RPN11 have significant prognostic value for HCC.

### 3.4. Genetic Alteration and Immune Infiltration Analyses of RPNs in HCC Patients

**Figure 6:**
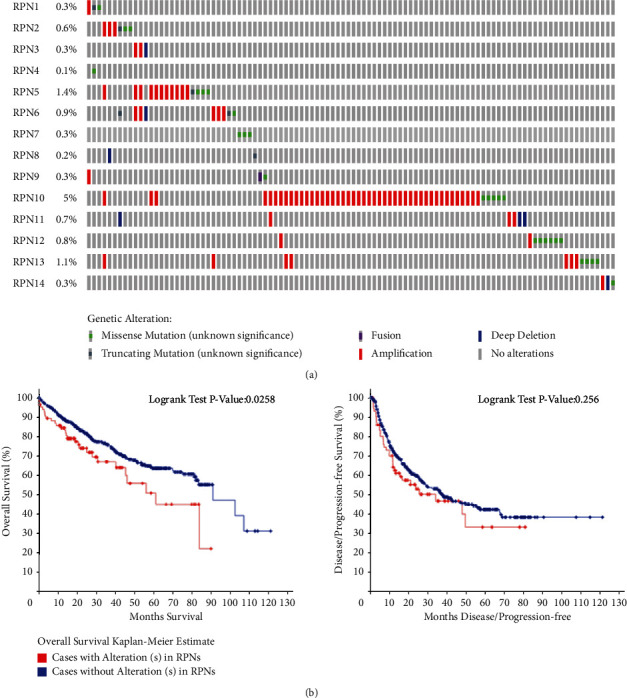
Genomic alterations of RPN of HCC (cBioPortal). (a) Oncoprint of RPNs' alteration in HCC. (b). Survival analysis of patients with or without mutation.

We used the cBioPortal online tool to analyze the genetic changes of RPNs in HCC. According to the TCGA dataset, the highest genetic variation rate in RPNs is RPN10 (5%), the lowest mutation rate is RPN4 (0.1%), and the others are RPN8 (0.2%), RPN14 (0.3%), RPN1 (0.3%), RPN3 (0.3%), and RPN7 (0.3%) (Figure 6(a)). Although changes are little in RPNs, those changes were correlated with overall patient survival (Figure 6(b)).

**Figure 7:**
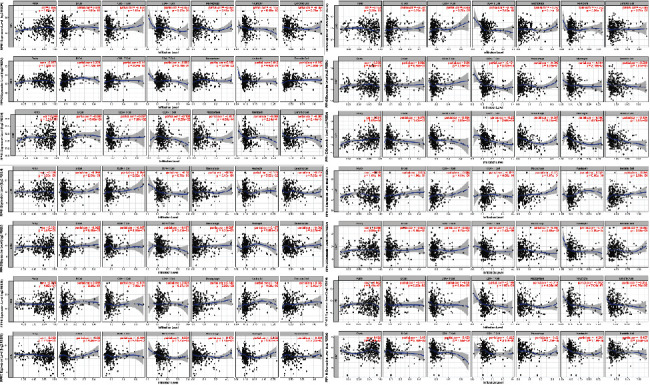
The correlation between the differently expressed RPNs and immune cell infiltration in HCC patients (TIMER).

The TIMER database is used to evaluate the relationship between the transcription level of RPNs and the level of immune infiltration in HCC. It was found that RPNs are involved in the inflammatory response and immune cell infiltration, which may affect the clinical outcome of HCC patients. The analysis results are shown in Figure 7. RPN2 expression was positively correlated with the infiltration of B cells, macrophages, and dendritic cells. RPN4 was negatively associated with the infiltration of CD4^+^ T cells. RPN5 was negatively correlated with the infiltration of CD4^+^ T cells while positively correlated with neutrophil infiltration. RPN6 expressions were positively associated with the infiltration of macrophages and neutrophils. RPN7 was negatively correlated with the infiltration of CD8^+^ T cells, CD4^+^ T cells, macrophages, neutrophils, and dendritic cells. RPN8 was negatively associated with the infiltration of purity and CD4^+^ T cells. RPN9 was negatively correlated with the infiltration of CD4^+^ T cells and neutrophils. RPN10 was positively correlated with the infiltration of purity cells while negatively correlated with CD8^+^ T cells, CD4^+^ T cells, macrophages, neutrophils, and dendritic cells. RPN11 was positively associated with the infiltration of neutrophils while negatively correlated with purity cells. RPN12 was positively correlated with the infiltration of CD4^+^ T cells. RPN13 was negatively correlated with B cells, CD8^+^ T cells, CD4^+^ T cells, macrophages, neutrophils, and dendritic cells. RPN14 was positively associated with the infiltration of purity cells while negatively with the B cells, CD8^+^ T cells, CD4^+^ T cells, macrophages, neutrophils, and dendritic cells. These results indicated that the level of RPNs' expression is associated with the level of immune infiltration in HCC.

### 3.5. The GGI and Functional Enrichment of RPNs

**Figure 8:**
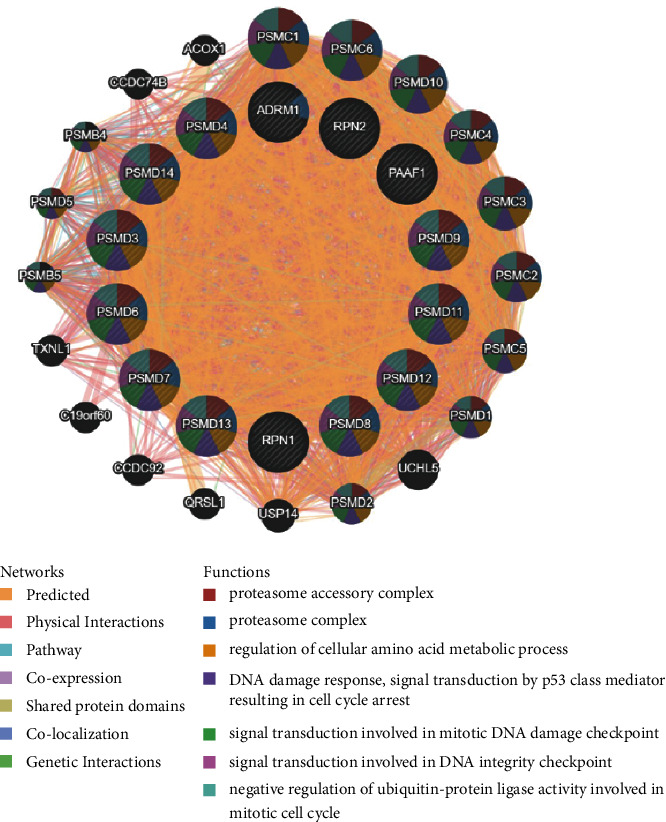
Gene-gene interaction network of differently expressed RPNs (GeneMANIA).

Identifying more details about RPNs could boost the understanding of their potential functions in HCC. Therefore, we constructed a GGI using the GeneMANIA database. In Figure 8, the 14 central nodes of RPNs are surrounded by 20 genes strongly associated with RPNs in physical interactions, co-localization, co-expression, prediction, genetic interactions, and pathways. The top five genes most associated are PSMC1 (proteasome 26S subunit, ATPase 1), PSMC6 (proteasome 26S subunit, ATPase 6), PSMD10 (proteasome 26S subunit, non-ATPase 10), PSMC4 (proteasome 26S subunit, ATPase 4), and PSMC3 (proteasome 26S subunit, ATPase 3). They all have genetic interactions and are correlated with functions such as proteasome accessory complex, proteasome complex, and regulation of the cellular amino acid metabolic process.

**Figure 9:**
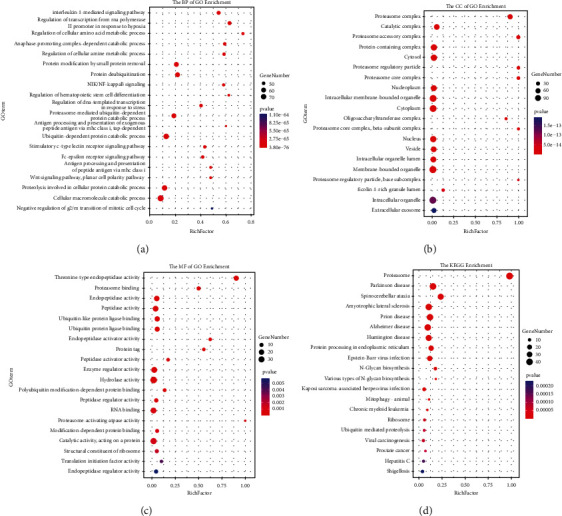
Predicted functions and pathways of RPNs. (BP, CC, MF, and KEGG) (String). (a). Bubble graph for GO BP enrichment. (b). Bubble graph for GO CC enrichment. (c). Bubble graph for GO MF enrichment. (d). Bubble graph for KEGG pathway enrichment.

As shown in Figure 9, besides protein binding, RNA binding, or ubiquitin-protein ligase activity, RPNs still play an important role in biosynthesis: extracellular exosome, translation initiation factor activity, and protein processing in endoplasmic reticulum activity.

### 3.6. RPN6 and RPN9 are Upregulated in HCC Tissues and Cell Lines

**Figure 10:**
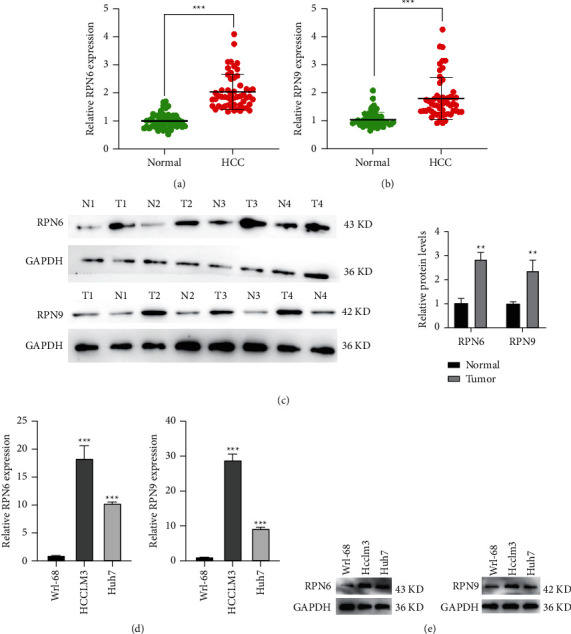
RPN6 and RPN9 are upregulated in HCC tissues and cell lines. (a). RPN6 mRNA expression was markedly elevated in HCC tissues relative to their normal counterparts determined by RT-qPCR. (b). RPN9 mRNA expression was markedly elevated in HCC tissues relative to their normal counterparts determined by RT-qPCR. (c). Western blot analysis was employed to confirm the expression of RPN6 and RPN9 in human HCC compared with the adjacent normal tissues. T tumors, N adjacent normal tissues. (d). RPN6 and RPN9 mRNA expression was markedly elevated in HCC cell lines than normal. (e). Western blot analysis was employed to confirm the expression of RPN6 and RPN9 in human HCC cell lines than normal.

Some RPNs' oncogenic roles in HCC were uncovered by other researchers [31–35]. To confirm our conclusions, we first conducted (RT-qPCR) to investigate the mRNA levels of RPN6 and RPN9 in 54 paired human HCC tissue specimens and their corresponding nontumorous tissue samples. The results showed that RPN6 and RPN9 mRNA expression was markedly elevated in HCC tissues relative to normal (Figures 10(a) and 10(b)). An upregulated protein expression levels of RPN6 and RPN9 were further confirmed in 4 paired specimens by immunoblotting assays (Figure 10(c)). To further investigate the oncogene function of RPN6 and RPN9, a panel of human HCC cell lines was evaluated by RT-qPCR and western blot. The PCR results showed that RPN6 and RPN9 were present at higher (Hcclm3 and Huh7) levels than Wrl-68. WB further confirmed an upregulated protein expression level of RPN6 and RPN9 in HCC cell lines.

Knockdown of RPN6 and RPN9 inhibits cell proliferation, migration, and invasion by the EMT process.

**Figure 11:**
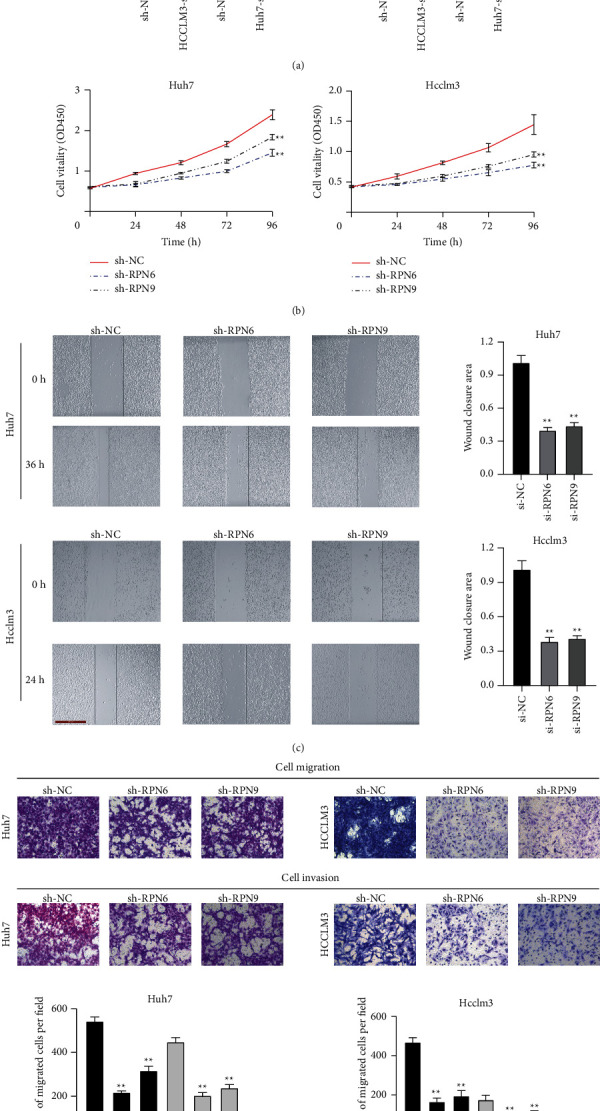
Knockdown of RPN6 and RPN9 suppresses the proliferation, migration, and invasion of HCC cells in vitro. (a). RT-qPCR analysis was employed to examine the efficiency of RPN6 and RPN9 knockdown. (b). CCK-8 assays result for RPN6 and RPN9 knockdown in Hcclm3 and Huh7 cells. (c). Wound-healing assay was performed to measure the migration ability of these cells. (d). Transwell assays were used to measure the migration ability of these cells.

**Figure 12:**
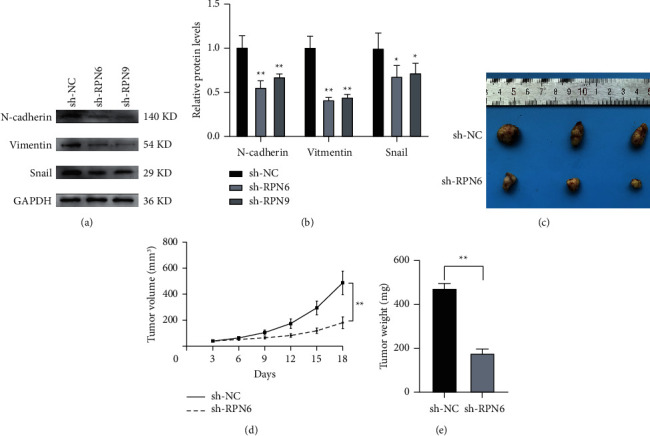
Knockdown of RPN6 and RPN9 inhibits cell proliferation, migration, and invasion by the EMT process. (a). N-cadherin, Snail, and Vimentin protein levels were detected in RPN6 or RPN9 stable knockdown by western blotting. (b). N-cadherin, Snail, and Vimentin protein levels were quantified in RPN6 or RPN9 stable knockdown. (c). Xenograft tumors were generated by injecting Hcclm3 cell-downregulated RPN6 or carrying a negative control vector. (d). The growth of xenograft tumors was measured by volume. (e). The tumor weight was recorded.

As RPN6 and RPN9 are upregulated in HCC tissues and cell lines, it is necessary to determine whether their suppression could affect the biological activity in HCC cells. We stably downregulated RPN6 and RPN9 in HCC cell lines, Hcclm3 and Huh7, using shRNA. As shown in Figure 11(a), RT-qPCR data indicated a significant loss of mRNA expression in those two HCC cell lines. The CCK-8 proliferation curves demonstrated that RPN6 and RPN9 knockdown remarkedly attenuated cell growth in Hcclm3 and Huh7 cells (Figure 11(b)). We further explored the potential impact of RPN6 and RPN9 knockdown on metastatic properties in HCC cells using wound scratch and transwell assays. As shown in Figure 11(c), the results showed that the loss function of RPN6 and RPN9 significantly suppressed the wound closure potential in Hcclm3 and Huh7 cells. In the transwell assay, attenuated RPN6 and RPN9 expression remarkably decreased migration and invasion capabilities in Hcclm3 and Huh7 cells. Thus, our results show that RPN6 and RPN9 expressions are essential for HCC cell proliferation, migration, and invasion. As epithelial-mesenchymal transition (EMT) markers are crucial in cell migration and metastasis, we investigated whether RPN6 and RPN9 affected the EMT process in HCC. As shown in Figure 12(a), western blot analysis documented that the silencing of RPN6 and RPN9 decreased the expression of mesenchymal markers (N-cadherin, Vimentin, and Snail) in Hcclm3 cells.

### 3.7. Knockdown of RPN6 Inhibits Tumor Growth in Vivo

To further validate whether RPN6 could affect tumor growth in vivo, the Hcclm3 cells with NC group or Sh-RPN6 group were inoculated subcutaneously into either side of nude mice. The mice were euthanized 18 days after injection. We found that tumors derived from Sh-RPN6 cells grew more slowly, and the final tumor weight was markedly lower than the NC group (Figures 12(c)–12(e)), which was consistent with the results in vitro.

## 4. Discussion

Hepatocellular carcinoma, featured by high morbidity and mortality, remains one of the most health threats to people worldwide [1]. Although surgical procedures have become the standard of treatment, most patients are not surgical candidates [4]. Therefore, to conquer HCC, screaming potential molecular biomarkers is a top priority.

By charging 80–90% of protein degradation, UPS is a sophisticated controlled system and responsible for many kinds of cellular procedures and oncogenesis [36, 37]. The Ribophorin family is an essential regulatory subunit of 26s proteasome. Some studies have reported that RPNs' deregulation has robust oncogene functions in multiple cancers [11–14]. Although some RPNs' oncogenic role in HCC has been proven, distinct roles of the RPN family in HCC remained ambiguous. Therefore, we performed a comprehensive bioinformatics analysis of RPNs in this study.

RPN1 is the largest proteasome subunit and functions as the recognition part of the ubiquitin-proteasome system proteins by the proteasome [38]. RPN1 was possessed by two adjacent regions designated as T1 and T2. T1 is the receptor site for specific UBL domain proteins, while T2 binds to USP14, a proteasome-associated deubiquitinating enzyme [39, 40]. As a crucial part of the UPS system, RPN1 was upregulated in lung and kidney cancer [41]. Abnormal RPN1/EVI1 fusion was also popular in myelodysplasia and acute myeloid leukemia [42] and could be one of the relevant genes to predict breast cancer prognostic significance [43]. In our study, data from the online datasets showed that RPN1 was more highly expressed in human HCC tissues than normal tissues. And RPN1 expression is related to tumor grade. Further, highly expressed RPN1 RNA was associated with a worse OS, which was not studied in the previous report.

Located at 20ql2-13.1, RPN2 is paralogous RPN1 and a highly conserved protein in the rough endoplasmic reticulum. In 2008, Takahashi et al. reported that overexpressed RPN2 is associated with docetaxel resistance by degenerating GSK3b in breast cancer [11]. Tominaga et al. found the other drug resistance function of RPN2 in breast cancer with a different target of CD63 [44]. Next, RPN2's oncogene role has been found in esophageal squamous cell carcinoma [12], osteosarcoma [13], gastric cancer [14], and HCC [32]. The Zhang et al. study showed that RPN2 might serve as a biomarker for colorectal cancer. Recently, Huang et al. identified that RPN2 was upregulated in HCC cell lines and HCC tissues. Overexpressed RPN2 expression increased cell proliferation, metastasis, invasion, and epithelial-mesenchymal transition [32]. RPN2 may precisely interact with STAT3 and NF-*κ*B pathways. Circ_0046599 also promoted HCC development by influencing RPN2 [33]. Moreover, RPN2 can be detected in blood exosomes [45] and urinary exosomes [46], expanding its application in diagnosis.

RPN6 is another essential subunit in the UPS system. Studies have proven that its high expression or phosphorylation may promote proteasome activity [47, 48]. RPN6 could protect pancreatic cancer cells from acute apoptosis [49]. In our work, we indicated that RPN6 was upregulated in HCC and its expression level is correlated with the patient's tumor grade and OS. Moreover, RPN6 is a sensitivity biomarker for HCC diagnosis. Previous studies thought RPN9 is an aging-related gene [50, 51]. Our study first revealed the oncogene role of RPN9 in cancers. Further work should be done to confirm our results.

Besides being an important subunit of the proteasome, RPN11 is also a robust proteasome deubiquitinase. Luo et al. reported that RPN11 plays an essential role in breast cancer progression [52]. Zhu et al. showed silencing RPN11 reduced the metastasis of esophageal squamous cell carcinoma [53]. Wang et al. indicted that RPN11 dysregulation may correlate with many cancers besides HCC [35]. On the expression level, the RPN11 we uncovered here might serve as an effective diagnostic and prognosis biomarker for HCC.

Our study showed that all members of the RPNs were significantly overexpressed in HCC, and their expression patterns were connected with the patient's tumor grade. The ROC curve suggested that RPN2, RPN3, RPN6, RPN9, RPN10, RPN11, and RPN12 have sensitivity and specificity in HCC diagnosis. Moreover, the expression of RPN1, RPN2, RPN5, RPN6, RPN9, and RPN11 was negatively correlated with patients' OS. Furthermore, the level of RPNs' expression is associated with the level of immune infiltration in HCC. The GGI and functional enrichment of RPNs were analyzed to predict their molecular functions and potential targets. Moreover, we performed western blot and qRT-PCR of RPN6 and RPN9 to verify these results. The silencing of RPN6 and RPN9 significantly influenced HCC cells' proliferation, migration, and invasion. An in vivo tumor model further validated the oncogene effect of RPN6 on HCC cell growth. Moreover, RPN6 and RPN9 could promote cell migratory and invasive potential by affecting the epithelial-mesenchymal transition (EMT) process. Our findings showed that the RPN family has the potential to be new prognostic and diagnostic markers and also drug targets for HCC. There are still some limitations in our study. Our findings required a multicenter, randomized, large clinical controlled trial, and the molecule processes behind RPNs should be addressed. As RPNs can be detected in blood and urine, the effect of liquid biopsy may be valued further. Despite the limitations above, our study first investigated the diagnostic and prognostic value of RPNs in HCC. Their results highlighted many RPNs have the potential to be new biomarkers and underlying targets for HCC.

## Figures and Tables

**Table 1:** Significant changes of RPNs' expression in the transcriptional level between cancer and normal tissues (oncomine database).

Gene ID	Types of HCC versus normal	Fold Change	*p* Value	t Test	References
RPN1	Hepatocellular Carcinoma versus Normal	1.249	1.09E^−14^	7.904	Roessler [26]
	Hepatocellular Carcinoma versus Normal	1.019	0.024	2.009	TCGA

RPN2	Hepatocellular Carcinoma versus Normal	1.713	1.54E^−74^	22.605	Roessler [26]
	Hepatocellular Carcinoma versus Normal	1.468	3.44E^−7^	5.927	Roessler [27]
	Hepatocellular Carcinoma versus Normal	1.022	3.27E^−4^	3.790	Guichard [28]
	Hepatocellular Carcinoma versus Normal	1.074	1.41E^−8^	6.045	TCGA
	Hepatocellular Carcinoma versus Normal	1.033	2.13E^−5^	4.248	Guichard [28]
	Hepatocellular Carcinoma versus Normal	1.465	0.012	2.546	Wurmbach [27]

RPN3	Hepatocellular Carcinoma versus Normal	1.231	0.001	3.074	Chen [29]
	Hepatocellular Carcinoma versus Normal	1.286	7.37E^−17^	8.617	Roessler [27]
	Hepatocellular Carcinoma versus Normal	1.046	0.001	3.165	TCGA
	Hepatocellular Carcinoma versus Normal	1.020	0.009	2.414	Guichard [28]

RPN4	Hepatocellular Carcinoma versus Normal	1.112	0.010	2.337	Chen [29]
	Hepatocellular Carcinoma versus Normal	1.239	7.50E^−11^	6.573	Roessler [26]
	Hepatocellular Carcinoma versus Normal	1.256	0.023	2.058	Roessler [26]

RPN5	Hepatocellular Carcinoma versus Normal	1.692	5.83E^−37^	13.892	Roessler [26]
	Hepatocellular Carcinoma versus Normal	1.110	2.46E^−9^	6.430	TCGA
	Hepatocellular Carcinoma versus Normal	1.042	9.93E^−7^	4.986	Guichard [28]]
	Hepatocellular Carcinoma versus Normal	1.034	0.005	2.778	Guichard [28]
	Hepatocellular Carcinoma versus Normal	1.153	0.020	2.062	Chen [29]

RPN6	Hepatocellular Carcinoma versus Normal	1.414	1.00E^−8^	5.881	Chen [29]
	Hepatocellular Carcinoma versus Normal	1.654	3.73E^−36^	13.732	Roessler [26]
	Hepatocellular Carcinoma versus Normal	1.407	0.011	2.571	Wurmbach [27]]
	Hepatocellular Carcinoma versus Normal	1.041	0.006	2.590	TCGA
	Hepatocellular Carcinoma versus Normal	1.011	0.024	1.997	Guichard [28]

RPN7	Hepatocellular Carcinoma versus Normal	1.465	1.02E^−23^	10.595	Roessler [26]

RPN8	Hepatocellular Carcinoma versus Normal	1.162	9.26E^−6^	4.329	Roessler [26]
	Hepatocellular Carcinoma versus Normal	1.373	0.014	2.375	Wurmbach [27]

RPN9	Hepatocellular Carcinoma versus Normal	1.458	4.73E^−29^	12.000	Roessler [26]
	Hepatocellular Carcinoma versus Normal	1.400	0.016	2.422	Wurmbach [27]

RPN10	Hepatocellular Carcinoma versus Normal	2.265	2.10E^−85^	25.343	Roessler [26]
	Hepatocellular Carcinoma versus Normal	2.533	1.20E^−11^	9.023	Wurmbach [27]
	Hepatocellular Carcinoma versus Normal	2.078	1.66E^−11^	9.550	Roessler [26]
	Hepatocellular Carcinoma versus Normal	1.097	9.63E^−7^	6.126	Guichard [28]
	Hepatocellular Carcinoma versus Normal	1.098	9.44E^−18^	10.186	Guichard [28]
	Hepatocellular Carcinoma versus Normal	1.318	2.06E^−18^	10.720	TCGA
	Hepatocellular Carcinoma versus Normal	1.340	8.84E^−5^	4.036	[30]

RPN11	Hepatocellular Carcinoma versus Normal	2.243	4.71E^−74^	22.195	Roessler [26]
	Hepatocellular Carcinoma versus Normal	2.061	3.84E^−7^	5.876	Roessler [26]
	Hepatocellular Carcinoma versus Normal	1.266	5.88E^−5^	3.941	Chen [29]
	Hepatocellular Carcinoma versus Normal	1.723	0.008	2.864	Wurmbach [27]

RPN12	Hepatocellular Carcinoma versus Normal	1.543	7.28E^−31^	12.413	Roessler [26]
	Hepatocellular Carcinoma versus Normal	1.477	0.003	2.928	Roessler [26]
	Hepatocellular Carcinoma versus Normal	1.148	0.007	2.507	Chen [29]
	Hepatocellular Carcinoma versus Normal	1.031	0.002	2.981	TCGA
	Hepatocellular Carcinoma versus Normal	1.015	0.033	1.854	Guichard [28]

RPN13	Hepatocellular Carcinoma versus Normal	1.044	3.63E^−5^	4.500	Guichard [28]
	Hepatocellular Carcinoma versus Normal	1.091	5.17E^−10^	6.763	TCGA
	Hepatocellular Carcinoma versus Normal	1.288	1.40E^−19^	9.407	Roessler [26]
	Hepatocellular Carcinoma versus Normal	1.371	0.002	3.094	Roessler [26]
	Hepatocellular Carcinoma versus Normal	1.174	0.007	2.487	Chen [29]
	Hepatocellular Carcinoma versus Normal	1.030	0.001	3.126	Guichard [28]

RPN14	Hepatocellular Carcinoma versus Normal	1.343	0.007	2.657	Wurmbach [27]
	Hepatocellular Carcinoma versus Normal	1.131	3.89E^−5^	3.994	Roessler [26]

## Data Availability

The transcription levels of RPNs and the changes of RPNs' expression between cancer and normal data were downloaded from the Oncomine database (https://www.oncomine.org) under the accession number (s): n8630, n8887, n8302, n4313, n8472, and n9158. The transcription levels of ITPRs between cancer and normal cells were downloaded from the UALCAN database (https://ualcan.path.uab.edu). Cancer patients' survival analysis data were downloaded from the Kaplan–Meier Plotter database (https://gepia.cancer-pku.cn). The ITPRs' gene expression data and clinic information were downloaded from the UCSC Xena database (https://xena.ucsc.edu/). The immune infiltrations data were downloaded from the Timer database (https://cistrome.shinyapps.io/timer/). The ITPRs' gene alteration data and clinic information were downloaded from the CbioPortal database (https://www.cbioportal.org). The gene relationships network data were downloaded from the GeneMANIA database (https://www.genemania.org). The methylation status of ITPRs' gene data was downloaded from the Wanderer database (https://maplab.imppc.org/wanderer). The GO and KEGG enrichment data were downloaded from the STRING database (https://string-db.org/).

## Supplementary Materials

Supplementary Materials Table S1. Primer sequences oligonucleotides information. Table S2. The ROC test results of RPN subunits.
