# Supplementary material for: RPNs Levels Are Prognostic and Diagnostic Markers for Hepatocellular Carcinoma
Source: J Oncol. 2022 Aug 29;2022:7270541. doi: 10.1155/2022/7270541 (PMC9444382; doi:10.1155/2022/7270541)
Supplement: Supplementary Materials — Table S1. Primer sequences oligonucleotides information. Table S2. The ROC test results of RPN subunits. [file 7270541.f1.pdf]

Table S1. Primer sequences oligonucleotides information.

Gene/miRNAs	Sequence
RPN6	Forward primer: 5′- GCGGAGGGATCGACAAATGG-3′<br>Reverse primer: 5′-TGGGACATAGCTTAAAGAGGCA-3′
RPN9	Forward primer: 5′-CACCTTGGGGTTAGTGGATGA-3′<br>Reverse primer: 5′-CTCGGTGTGGTTCCCTTGT -3′
GAPDH	Forward primer: 5′- ATTTGCCTGCATTACCGGTC-3′<br>Reverse primer: 5′-ATCAACGTTTTCTTTTCGG-3′
RPN6-siRNA	Forward primer: 5′-CAAGAAAUUUCAUGGGAUUTT-3′<br>Reverse primer: 5′-AAUCCCAUGAAAUUUCUUGTT -3′
RPN9-siRNA	Forward primer: 5′-AGGCAGUGAUCCUGUGUAATT-3′<br>Reverse primer: 5′-UUACACAGGAUCACUGCCUTT -3′

Table S **2 The ROC test results of RPN subunits.**

Gene ID	Area	*P* Value	95% Confidence Interval	
			Lower Bound	Upper Bound
RPN1	0.494	0.898	0.421	0.568
RPN2	0.818	0.000	0.768	0.867
RPN3	0.728	0.000	0.676	0.780
RPN4	0.593	0.032	0.527	0.660
RPN5	0.349	0.001	0.275	0.423
RPN6	0.704	0.000	0.650	0.759
RPN7	0.438	0.151	0.372	0.503
RPN8	0.510	0.824	0.454	0.566
RPN9	0.650	0.001	0.585	0.715
RPN10	0.880	0.000	0.841	0.918
RPN11	0.815	0.000	0.765	0.864
RPN12	0.620	0.006	0.556	0.683
RPN13	0.593	0.032	0.521	0.665
RPN14	0.553	0.226	0.480	0.625
